# Sustainable *Hibiscus rosa-sinensis*-templated MnO_2_@TiO_2_ nanocomposites for the visible-light-driven photocatalytic degradation of hazardous organic dyes

**DOI:** 10.1039/d6ra03027k

**Published:** 2026-07-03

**Authors:** Yashneeti Mehta, Sonal Chauhan, Dinesh Kumar Arya, Parul Khurana, Nitesh Kumar, Sheenam Thatai

**Affiliations:** a Amity Institute of Applied Sciences, Amity University Noida U.P. 201313 India sthatai@amity.edu; b Aacharya Narendra Dev College, University of Delhi New Delhi 110019 India; c Khalsa College of Arts, Science and Commerce, Mumbai University Mumbai 400019 India; d Amity Institute for Advanced Research and Studies (Material & Devices), Amity University Noida U.P. 201313 India

## Abstract

The persistent presence of organic dyes poses a significant modern challenge, prompting scientific efforts to develop materials capable of mitigating their harmful effects on our primary life source, water. Here, we present the biosynthesis of titanium dioxide nanoparticles using the aqueous flower extract of the *Hibiscus rosa-sinensis* plant by a microwave-assisted method. Initially, we synthesized titanium dioxide nanoparticles for the photocatalytic degradation of methyl orange and methylene blue, which show degradation efficiencies of only 57.4849% and 70.55%, respectively. Their poor photocatalytic activity under visible light necessitated the incorporation of manganese dioxide to make manganese-implanted titania NCs. Surface modification with manganese dioxide highly increases the degradation efficiency for methyl orange and methylene blue, which is 85% and 95.51%, respectively, under visible light. A detailed characterization was performed using ultraviolet visible spectroscopy, Fourier-transform infrared spectroscopy, X-ray diffraction, scanning electron microscopy, dynamic light scattering, transmission electron microscopy, Brunauer–Emmett–Teller surface analysis, photoluminescence spectroscopy and electrochemical studies. The synthesized nanocomposite showed biological properties, similar to antibacterial and antioxidant properties, as a secondary application, due to the generation of ROS species, which also photocatalytically degraded the dyes effectively. The practical stability of the composite was confirmed by catalyst reusability over three successive cycles. This shows that the biosynthesized manganese dioxide with titanium dioxide is an excellent and versatile catalyst for wastewater treatment.

## Abstract 2

*Hibiscus rosa-sinensis* assisted synthesis of MnO_2_@TiO_2_ nanocomposites enables efficient visible-light photocatalytic degradation of methylene blue (95%) and methyl orange (89%).

## Graphical abstract

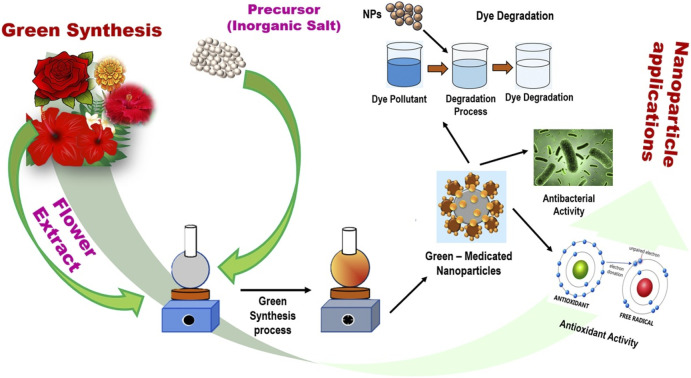

## 1. Introduction

Sustainable waste management and conversion processes are being crucially performed by nanotechnology. Since high surface area, tunable reactivity, and catalytic activity are exhibited by nanoparticles, environmental wastes can be tackled more effectively with these materials than with conventional materials. By working at the nanoscale, waste pollutants can interact with these materials at the molecular level, accelerating decomposition or facilitating conversion into harmless compounds. The development of waste-derived nanomaterials is given as a striking example; agricultural and municipal wastes have been used as precursors for synthesizing NPs so that they can be reused for environmental applications.^1^ By using these green synthesis methods, waste management is moving towards a circular model, where the production of cleanup technologies is fed by waste itself. Significant environmental challenges are posed by dyes, which are often used in industrial sectors, when they accumulate in wastewater. Adverse health effects are caused by these toxic dyes on aquatic as well as human health. Due to the presence of heavy metals and carcinogens, these dyes affect neurological health as well as delay development. To mitigate the environmental impact of dye pollution, industries, regulatory authorities, and scientific communities need to collaborate.^1^ Dye molecules can be removed from wastewater using physical methods, like filtration, adsorption, coagulation, flocculation, membrane processes, electrocoagulation, and biological methodologies.^2,3^ For removing organic dyes from wastewater, photocatalytic degradation is a promising method. It is a very environment-friendly and cost-effective methodology along with low energy consumption in the removal of organic dyes.^4,5^

Nanotechnology goes with the principles of sustainability by often reducing the need for toxic chemicals or extreme conditions in waste processing. Nearly one-third of the total solid waste generated can be responsible for floral waste. A cascade of environmental issues is caused by the improper floral waste disposal, such as water contamination, impurity of air, and greenhouse gas emissions, along with public health concerns. An emerging solution is to utilize flower waste as a raw material to synthesize nanomaterials, mainly for photocatalytic applications. Multiple advantages are offered by this approach as we manage both waste management and materials production, which are carried out in a sustainable manner. This reduces not only the waste disposal problem, but also the need for toxic chemical reagents usually used in nanoparticle synthesis.^6^ A high number of bioactive compounds such as polyphenols, flavonoids, terpenoids, and reducing sugars are found in flower extracts, and natural reducing and capping agents are acted upon by these in the green synthesis of nanoparticles.^7^ The potential of *Hibiscus rosa-sinensis* flower has been explored for the synthesis of metal NPs. The metals are converted into NPs with the help of reducing as well as stabilizing agents like flavonoids, phenols and tannins, which are present in the flower extract.^8^ Due to the presence of antioxidant properties in these compounds, oxidative damage and scavenging of free radicals does not take place. The extracts are also used in various biomedical as well as environmental applications due to their antioxidant and anti-inflammatory properties.^6,9^

**Fig. 1:**
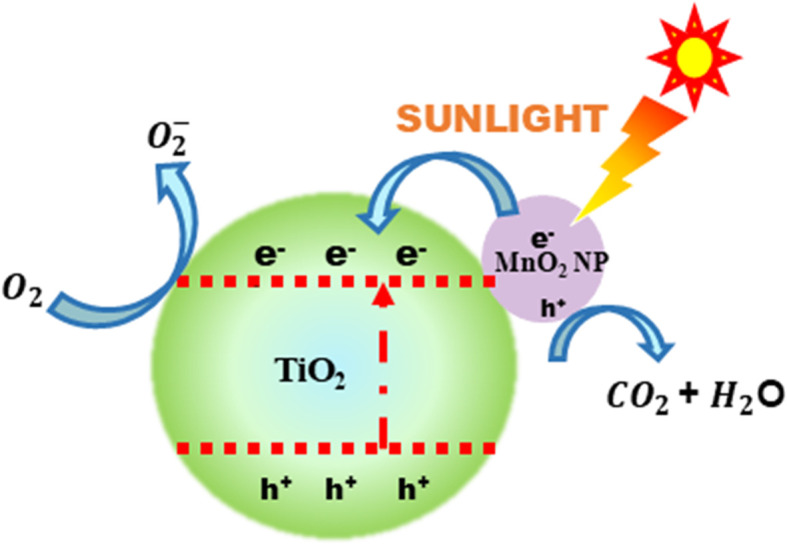
Schematic of the photocatalytic degradation of organic pollutants by the MnO_2_@TiO_2_ NCs under solar radiation.

Due to the low production costs and mechanical and chemical stabilities, TiO_2_ nanostructures are widely used in various industries. Pure TiO_2_ is very beneficial for photovoltaic as well as photocatalytic applications because it exhibits good catalytic ability and electron mobility.^10^ However, due to its wide bandgap energy, TiO_2_ has low photocatalytic activity under visible light.^11^ The incorporation of metals like Mn, Fe, Co, Cu, Ni and Cr can help overcome this and can also enhance the photocatalytic activity.^12^ These metals can produce an impurity level in the TiO_2_ bandgap, which will decrease the bandgap along with the shifting of absorption towards a longer wavelength. Metal ions like MnO_2_^+^, PT^2+^, Au^3+^, and Mn^4+^ can act as an electron sink. They capture photogenerated electrons and reduce charge recombination, due to which reactive oxygen species (ROS) are generated. The photocatalytic activity can also be increased through the surface plasmon resonance (SPR).^13,14^ Transition metals like manganese have shown promise in extending the optical response of TiO_2_ into the visible region by creating intermediate energy levels within the band structure, as shown in Fig. 1.

Manganese oxide (MnO_2_) is a stable and cost-effective material with high specific surface area and mild oxidation performance.^15,16^ The presence of MnO_2_ in TiO_2_ NCs improves the optical and photocatalytic properties compared to pristine TiO_2_ NCs. The photocatalytic breakdown of organic materials in organic wastewater is aided by TiO_2_@MnO_2_ NCs and solar light. The reduction of superoxide anions by electrons results in the creation of hydroxyl radicals, which break down organic contaminants and mineralize into CO_2_ and H_2_O, as shown in Fig. 1.

The modification of TiO_2_ NPs with transition metal oxides such as MnO_2_ for enhanced visible light photocatalysis has been widely reported. Conventional chemical synthesis routes for MnO_2_@TiO_2_ NCs typically require toxic reducing agents, high-pressure autoclaves, or multi-step processing. However, the phytochemicals present in the flower extract simultaneously serve as reducing, capping, and stabilizing agents under mild conditions in plant extract-assisted green synthesis routes, offering a sustainable alternative. The phytochemicals anthocyanins, flavonoids, phenolics, and terpenoids are known for their strong metal-chelating and reducing capabilities found in the *Hibiscus rosa-sinensis* flowers. In this work, *Hibiscus rosa-sinensis* flower extract acts as a multifunctional green reagent for the sol–gel synthesis of MnO_2_@TiO_2_ NCs. They break the co-chelation of Ti^4+^ and Mn^2+^ precursors by the same phytochemicals to promote close phase integration at the molecular level.^17,18^

## 2. Experimental

### 2.1. Materials

*Hibiscus rosa-sinensis* flowers, also known as hibiscus flowers, were collected from the botanical premises of Amity University, Noida, India. Manganese(ii) sulfate monohydrate (MnSO_4_·H_2_O) was obtained from Sigma-Aldrich, tetraethyl orthotitanate (TEOT) was obtained from Sigma-Aldrich, and denatured ethanol (>99%), MB, and MO orange dye were obtained from Sigma-Aldrich.

### 2.2. Extract preparation

*Hibiscus rosa-sinensis* (commonly known as the hibiscus flower or China rose) were collected from the botanical premises of Amity University, Noida, India. We thoroughly cleaned the flower petals with double-distilled water to eliminate surface impurities and contaminants. Subsequently, the cleaned petals were air-dried under direct sunlight until completely dehydrated. Then we ground the dried material into a fine powder using a sterilized mortar and pestle, followed by sieving to ensure uniform particle size distribution and surface area.^19^

Then, 10 g of the powdered flower material was mixed with 100 mL of double-distilled water in a 250 mL borosilicate beaker for the extraction process. The mixer was subjected to microwave-assisted extraction for 2 min at 400 W to enhance phytochemical release. At room temperature, the solution, which was prepared earlier by microwaving, is allowed to cool down.

Whatman no. 4 filter paper was used for filtering the extract. For the removal of unwanted matter and for ensuring maximum clarity, the process of filtration was repeated four times. The final extract was stored in an amber glass container and refrigerated at 4 °C until use in the synthesis of NPs.

### 2.3. Biosynthesis of TiO_2_ NPs

To synthesize the TiO_2_ NPs, we use the sol–gel method. First, 20 mL of C_2_H_5_OH was taken in a round-bottom flask into which 3 mL of TEOT was added under continuous stirring at room temperature to obtain a homogeneous mixture. Slowly, we will add 20 mL of freshly prepared flower extract dropwise with the help of a dropper in the mixture, and the stirring is done so that the phytochemicals present in the flower extract can reduce as well as stabilize the solution.

After that, the solution was stirred continuously for 3 h, so that the reaction occurred completely along with the formation of uniform particles. After the reaction was completed, the solution was centrifuged to obtain the precipitate, which was then washed with Millipore water and C_2_H_5_OH thoroughly, so that if there are any organic residues or unreacted precursors, they will be removed.

The derived precipitate was then oven-dried at 60 °C for removing the moisture content and then calcined at 500 °C for another 3 h in a muffle furnace to obtain pure TiO_2_ NPs for further experiments as well as to increase their shelf life.

### 2.4. Synthesis of MnO_2_@TiO_2_ NCs

**Fig. 2:**
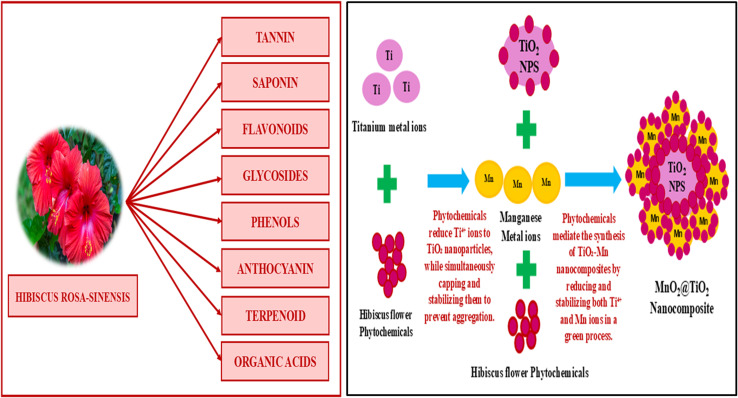
(Left) Phytochemical constituents of the *Hibiscus rosa-sinensis* flower extract. (Right) Schematic of the green sol–gel synthesis of the TiO_2_ NPs and MnO_2_@TiO_2_ NCs mediated by the *Hibiscus* flower phytochemicals.

MnO_2_@TiO_2_ NCs are also synthesized using the sol–gel technique. In this process, the solution of MnSO_4_·H_2_O was prepared by adding 0.003 M of MnSO_4_·H_2_O in 100 mL of double-distilled water and stirred continuously to obtain a homogeneous mixture. From the stock solution, 40 mL is taken and transferred to a round-bottom flask. Then, 0.5 g of the earlier synthesized TiO_2_ was added with constant stirring at 60 °C. After that, 20 mL of freshly prepared flower extract was added dropwise to the reaction mixture with the help of a dropper and stirred continuously. Both the reducing and stabilizing agents acted upon the flower extract. The reaction mixture was stirred for 2 h at 60 °C for the formation of the nanocomposite. After the reaction was completed, the solution was again subjected to centrifugation, and the obtained precipitate was washed with double-distilled water and C_2_H_5_OH thoroughly to get rid of unwanted residues or phytochemicals. Fig. 2 shows the mechanism of the above-mentioned process.

The washed precipitate was then oven-dried at 60 °C and then calcined for 2 h at 300 °C to obtain the final MnO_2_@TiO_2_ NCs.^20^

### 2.5. Characterization details

The optical absorption properties of the solid powder sample of TiO_2_ and MnO_2_@TiO_2_ NCs were examined using an Agilent Cary 5000 doubles out-of-plane monochromator in the spectral range of 200 to 800 nm. A PerkinElmer spectrometer was used to record the FT-IR spectra for the determination of surface functional groups. A Nano Plus/Particulate System DLS was used for determining the particle size and particle distribution of the synthesized TiO_2_ NPs and MnO_2_@TiO_2_ NCs in an aqueous suspension. A Bruker D2 phaser was used for analyzing the crystalline structure, along with the phase composition of the biosynthesized materials, by X-ray diffraction (XRD) with Cu Kα radiation (*λ* = 1.54184 Å) at 40 kV and 40 mA. The electrochemical behaviour of TiO_2_ NPs and MnO_2_@TiO_2_ NCs was investigated by cyclic voltammetry, Linear Sweep Voltammetry and Electrochemical Impedance Spectroscopy using a Metrohm. We used glassy carbon for drop-casting along with Ag/AgCl as an electrode and the electrolyte used was 0.5 M H_2_SO_4_. We examined the surface form and microstructure of the biosynthesized TiO_2_ and MnO_2_@TiO_2_ NCs using Scanning Electron Microscopy (SEM). The TEM images of the phyto-synthesised TiO_2_ NPs and MnO_2_@TiO_2_ NCs provided information about the morphological characteristics.

### 2.6. Experimental setup for dye degradation studies

We calculated the photocatalytic activity of the synthesized materials by observing the degradation of two organic dyes, MO and MB under visible light conditions. Initially, the degradation performance of the biosynthesized TiO_2_ NPs was assessed. However, the removal efficiency was found to be relatively low, indicating limited photocatalytic activity. ^21–23^

To enhance the catalytic performance, MnO_2_-loaded TiO_2_ NCs (MnO_2_@TiO_2_ NCs) synthesized *via* a green route were employed as the catalyst. The photocatalytic experiments were conducted in a custom-built photoreactor equipped with a visible light source (*λ* > 400 nm) and a magnetic stirrer to ensure uniform irradiation and suspension of the photocatalyst. In each experiment, a known concentration of dye solution (1 mg mL^−1^) was mixed with a fixed dose of catalyst (*e.g.*, 50 mg per 50 mL solution), and we stirred the mixture in the dark for 60 minutes to start adsorption–desorption equilibrium on the catalyst surface.

Subsequently, we irradiated the dye-catalyst suspension under visible light and withdrew portion (typically 3 mL) at regular time intervals. These samples were immediately centrifuged to remove particulate matter, and the clear supernatant was analysed using a UV-vis spectrophotometer. To monitor the degradation kinetics, the decrease in the characteristic peak's absorbance for MO (*λ*_max ≈ 464 nm) and MB (*λ*_max ≈ 665 nm) was recorded over time. From the UV-visible absorption spectra of an aliquot amount of dye solution, the efficiency of the degradation of given dyes were given by eqn (1) as follows:1
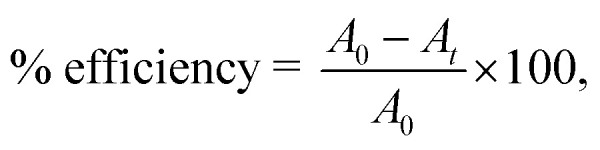
where *A*_0_ is used to represent the initial absorbance of the dye solution and *A*_*t*_ is used for the absorbance at time ‘*t*’.

### 2.7. Antioxidant study

The antioxidant potential of the synthesized MnO_2_@TiO_2_ NCs and TiO_2_ NPs was quantitatively evaluated using the 2,2-diphenyl-1-picrylhydrazyl (DPPH) free radical scavenging assay, and this method is widely accepted for evaluating the radical neutralization potential of nanomaterials.^24^ We freshly prepared a 0.1 mM DPPH solution by dissolving the required amount of DPPH in 100 mL of analytical-grade CH_3_OH. We will then store the solution in the dark for 30 min at room temperature to minimize spontaneous photodegradation and to ensure stabilization of the free radicals before use.

For the assay, we mixed 2 mL of the prepared DPPH solution with 1 mL of the test dispersions containing either MnO_2_@TiO_2_ NCs or pure TiO_2_ NPs. The test dispersions were formulated by sonicating the nanomaterials in CH_3_OH at a concentration of 1 mg mL^−1^, respectively, to ensure a homogeneous suspension. The mixtures were vortexed briefly and incubated in the dark for an additional 30 min at room temperature to allow for complete interaction between the DPPH radicals and the antioxidant-active surface of the nanomaterials. The reaction was carried out in a triplet and the mean is taken.

The mechanism reflects antioxidant activity by using the absorbance spectrophotometrically at 517 nm using a UV-vis spectrophotometer. A blank sample containing only CH_3_OH and DPPH was used as a control. The scavenging efficiency (%) was calculated based on the decrease in absorbance relative to the control, following eqn (2):2
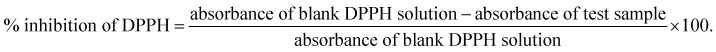


### 2.8. Antibacterial study

We investigated the antibacterial efficacy of the synthesized MnO_2_@TiO_2_ NCs and TiO_2_ NPs against a Gram-negative bacterial strain, *Escherichia coli* (*E. coli*) using the agar well diffusion method. The assay was conducted at a concentration of 5 µg mL^−1^ of the test material, to assess the dose-dependent antibacterial activity. We will now inject the fresh cultures of *E. coli* onto Mueller–Hinton agar (MHA) plates using a sterile cotton wipe to create a uniform bacterial region. We then cleanly made wells of approximately 4 mm diameter into the agar, and then, added 50 µL of each nanoparticle dispersion (prepared in sterile Millipore water) into the respective wells. At 37 °C for 24 h under visible light, the plates were incubated.

### 2.9. Scavenging activity

The most common reactive species involved in dye degradation was determined, and radical scavenging studies were carried out under identical photocatalytic conditions. To achieve adsorption–desorption equilibrium, 50 mg of MnO_2_@TiO_2_ NCs was mixed with 50 mL of dye solution (1 mg mL^−1^) and swirled in the dark for 60 min. The combination was irradiated with visible light (*λ* > 400 nm) in a custom-built photoreactor with a magnetic stirrer. Prior to irradiation, scavengers were introduced at appropriate concentrations. EDTA for photogenerated holes, isopropanol (IPA) for hydroxyl radicals (˙OH), as well as *p*-benzoquinone (BQ) for superoxide radicals (˙O_2_^−^) were included.

## 3. Results and discussion

### 3.1. Optical and chemical characterization

**Fig. 3:**
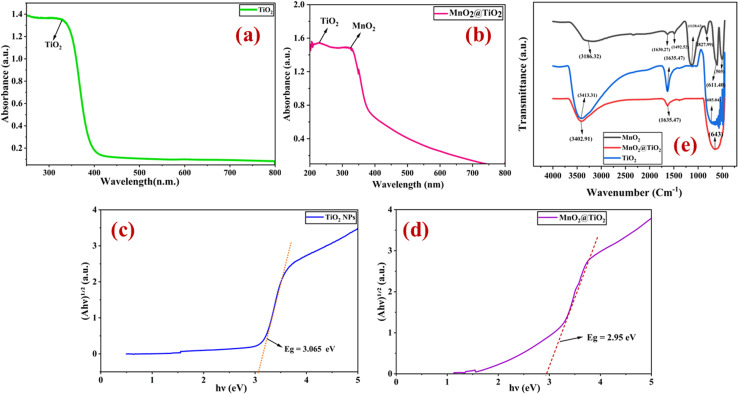
Optical characterization. UV-vis absorption spectra of the (a) TiO_2_ NPs and (b) MnO_2_@TiO_2_ NCs. Tauc plots of the (c) TiO_2_ NPs (*E*_g_ = 3.065 eV) and (d) MnO_2_@TiO_2_ NCs (*E*_g_ = 2.95 eV). (e) FTIR spectra of MnO_2_, MnO_2_@TiO_2_ NCs, and TiO_2_ NPs.

We studied the optical properties of TiO_2_ NPs and MnO_2_@TiO_2_ NCs using UV-visible diffuse reflectance spectroscopy (UV-DRS) and observed the corresponding spectral changes shown in Fig. 3(a) and (b). A sharp, single absorption edge at approximately 380 nm, which is characteristic of the band-to-band transition in anatase TiO_2_ NPs with negligible visible light absorption, is displayed by the UV-DRS spectrum of bare TiO_2_ NPs. In contrast, two distinct absorption features are revealed by the UV-DRS spectrum of MnO_2_@TiO_2_ NCs. The spectrum shows one peak corresponding to TiO_2_ NPs at ∼230 nm and a second shoulder at ∼330 nm, which are attributed to the d–d electronic transitions and charge transfer transitions characteristic of MnO_2_.^25,26^ Additional optical evidence for the coexistence of both TiO_2_ NPs and MnO_2_ phase is provided by the presence of both absorption features in the composite spectrum, which is fully consistent with XRD and FTIR results. Furthermore, an enhanced visible light harvesting capability of the composite is indicated by the absorption onset of MnO_2_@TiO_2_ NCs extending further into the visible region compared to bare TiO_2_ NPs.

We determined the optical bandgap energies from the Tauc plots (*Ahν*)^1/2^*vs. hν* for indirect bandgap semiconductors by extrapolating the linear portion of the absorption edge to the *x*-axis. We determined the bandgap of the TiO_2_ NPs to be *E*_g_ = 3.065 eV, which closely agrees with the reported value for anatase TiO_2_ NPs, as shown in Fig. 3(c) and (d), and confirms the phase purity established by XRD. The study shows that the bandgap of MnO_2_@TiO_2_ NCs is 2.95 eV, a 0.115 eV reduction compared to bare TiO_2_ NPs. The electronic interaction between MnO_2_ and TiO_2_ at the heterojunction interface causes this bandgap narrowing, because the introduction of MnO_2_ creates new intra-bandgap energy states that reduce the effective optical transitional energy. Enhanced photocatalytic activity of the composite under visible light irradiation is directly caused by the reduced bandgap of MnO_2_@TiO_2_ NCs, which enables excitation by longer-wavelength visible light photons that bare TiO_2_ NPs cannot activate.

FTIR spectroscopy identified the functional groups and chemical bonding environments in MnO_2_, TiO_2_, and MnO_2_@TiO_2_ NCs, revealing changes in the spectra shown in Fig. 3(e). Characteristic absorption bands at 505 and 611 cm^−1^ (attributed to the Ti–O–Ti stretching vibrations),^27^ a band at 643 cm^−1^ (corresponding to Ti–O stretching), a band at 1635 (from absorbed water O–H bending), and a broad band cantered at ∼3413 cm^−1^ (from surface hydroxyl O–H stretching) are displayed in the FTIR spectrum of bare TiO_2_ NPs. A broad O–H band at 3186 cm^−1^ indicative of Mn–OH surface groups, along with bands at 1492, 1128, and 827 cm^−1^ assignable to Mn–O stretching and bending vibrations, is shown by the FTIR spectrum of MnO_2_. Notable modifications are exhibited in the FTIR spectrum of MnO_2_@TiO_2_ NCs rather than it being a simple superposition of the individual component spectra. The simultaneous presence of both oxide phases is confirmed by the coexistence of both Ti–O (at 611 cm^−1^) and Mn–O vibrational features in the composite spectrum, consistent with XRD and EDX results. Modified hydrogen bonding environment at the interface suggests the Mn–O–Ti interfacial bond formation, which is indicated by the shift of the broad O–H stretching band in MnO_2_@TiO_2_ NCs to approximately 3402 cm^−1^ relative to both pure TiO_2_ (3413 cm^−1^) and MnO_2_ (3186 cm^−1^). Electronic coupling between the MnO_2_ and TiO_2_ phases is enhanced by this interfacial bonding as the main structural property, which increases the charge transfer across the heterojunction.

### 3.2. Morphological and structural characterization

**Fig. 4:**
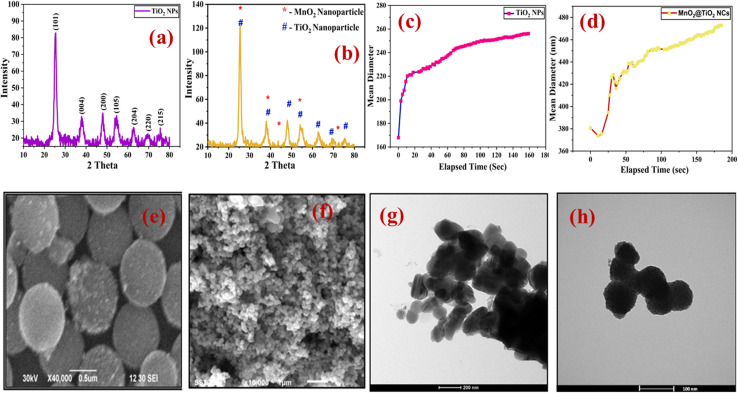
Structural and morphological characterization. PXRD patterns of the (a) TiO_2_ NPs and (b) MnO_2_@TiO_2_ NCs. DLS particle size distributions of the (c) TiO_2_ NPs and (d) MnO_2_@TiO_2_ NCs. SEM images of the (e) TiO_2_ NPs and (f) MnO_2_@TiO_2_ NCs. TEM images of the MnO_2_@TiO_2_ NCs at a scale of (g) 200 nm and (h) 100 nm.

Fig. 3 presents the PXRD patterns of the TiO_2_ NPs and MnO_2_@TiO_2_ NCs. All diffraction peaks of TiO_2_ NPs at 2*θ* = 25.30°, 37.98°, 47.97°, 54.53°, and 62.76° index exclusively to the anatase phase (JCPDS 21-1272), confirming phase–pure anatase TiO_2_ with no rutile or brookite impurities, as shown in Fig. 4(a) and (b). The crystallite size calculated from the dominant (101) reflection (*d* = 3.5200 Å) using the Scherrer eqn (3) is 7.36 nm:^28^3
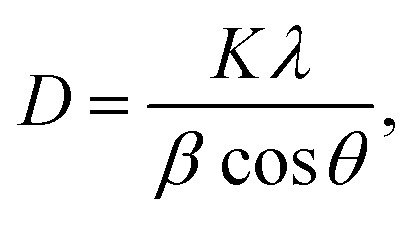
where *D* is the crystallite size, *K* is the Scherrer constant, *λ* is the X-ray wavelength, *β* is the FWHM in radius, and *θ* is the Bragg angle in radians.

The retention of all anatase TiO_2_ reflections, alongside additional peaks assigned to β-MnO_2_ pyrolusite (JCPDS 24-0735), confirms the successful formation of the MnO_2_@TiO_2_ NCs. Direct crystallographic evidence of interfacial lattice strain at the MnO_2_–TiO_2_ interface is provided by a systematic contraction in the TiO_2_ (101) *d*-spacing from 3.5200 Å to 3.5064 Å. Genuine electronic coupling between the two phases is confirmed by this shift, which is caused by the ionic radius mismatch between Mn^4+^ (0.53 Å) and Ti^4+^ (0.605 Å).^29^ An increase in the TiO_2_ crystallite size to 8.28 nm is observed modestly in the composite, while an average size of 12.11 nm is exhibited by the β-MnO_2_ crystallites.^30^

Our findings are confirmed by SEM analysis (Fig. 4). Smooth, well-defined spherical particles are displayed by the pure TiO_2_ NPs, while significantly roughened surfaces with uniformly distributed granular features, corresponding to the deposited MnO_2_ nanoparticles, are exhibited by the MnO_2_@TiO_2_ NCs, which are dimensionally consistent with a Scherrer-calculated MnO_2_ crystallite size of 12 nm, as shown in Fig. 4(e) and (f). The polycrystalline nature of the primary particles is confirmed by the larger particle dimensions observed in SEM relative to the Scherrer crystallite sizes. Together, the successful formation of closely interfaced MnO_2_@TiO_2_ NCs is collectively and consistently established by the *d*-spacing contraction dual-phase PXRD pattern along with SEM surface roughening.

The hydrodynamic diameter and size distribution of TiO_2_ NPs and MnO_2_@TiO_2_ NCs synthesised by the flower extract were determined by DLS analysis. The average hydrodynamic diameter is found to be 256 nm of the TiO_2_ NPs with monomodal distribution. Upon doping with MnO_2_ NPs, the sizes increase to 472 nm as compared to TiO_2_ NPs, which shows that MnO_2_@TiO_2_ NCs have been successfully formed.^31^ The increases in size may be due to the deposition integration of MnO_2_ over the TiO_2_ matrix, as shown in Fig. 4(c) and (d).

We examined the surface morphology of TiO_2_ NPs and MnO_2_@TiO_2_ NCs using SEM. Well-defined, smooth surface spherical particles with a relatively uniform size distribution are revealed to be of pure TiO_2_ NPs by the SEM images. Considerably rougher surface textures and small particles, which are uniformly distributed across the particle surfaces, distinguish the MnO_2_@TiO_2_ NCs in sharp difference. Direct morphological evidence for the successful deposition of MnO_2_ nanostructures onto the TiO_2_ NPs surface is provided by this transformation from smooth spherical TiO_2_ surfaces to rough, small particle-modified composite surfaces, consistent with the XRD findings that identified both phases in the composite.

This surface-modified structure further demonstrates the TEM analysis of the MnO_2_@TiO_2_ NCs. Spherical primary particles in the size range of approximately 50–150 nm with regions of higher electron density (darker shade) at the particle periphery and inter-particle junctions are revealed by the TEM images consistent with the presence of a denser MnO_2_ phase coating the TiO_2_ core.^32^ The drying of the sample during grid preparation and the intrinsic tendency of the NC system towards agglomeration cause the clustering of particles observed in the TEM image, as shown in Fig. 4(g) and (h).

### 3.3. Elemental and surface characterization

**Fig. 5:**
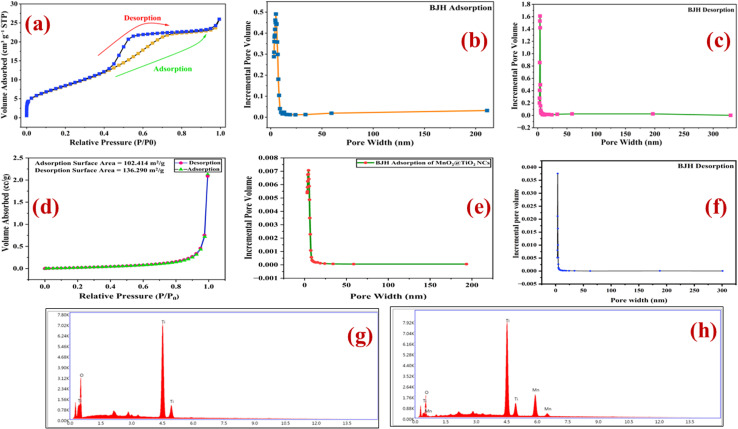
Surface and elemental characterization. N_2_ adsorption–desorption isotherms of the (a) TiO_2_ NPs and (d) MnO_2_@TiO_2_ NCs. BJH adsorption and desorption pore size distributions of the TiO_2_ NPs (b and c) and MnO_2_@TiO_2_ NCs (e and f). EDX spectra of the (g) TiO_2_ NPs and (h) MnO_2_@TiO_2_ NCs.

The main constituent elements are titanium and oxygen, which is confirmed by the EDAX spectrum with a weight percentage of 61.86% of titanium along with 38.14% of oxygen, given in Table 1. The spectrum shows the absence of any other additional element, which showcases the purity of the nanomaterial. We conclude the green synthesis of MnO_2_@TiO_2_ NCs by the EDAX spectrum, which shows that the composite contains Ti, Mn and O, which do not contain any impurities. The presence of Mn confirms the successful doping of Mn on the TiO_2_ NP surface. The percentage of Ti present in the composite indicates that TiO_2_ is the most dominating phase. The elemental composition is Ti (50.72 wt%), Mn (22.19 wt%) and O (27.09%), as given in the elemental composition in Table 2, derived from eZAF quantification, as shown in Fig. 5(g) and (h).

**Table 1:** eZAF SmartQuant results for TiO_2_

Element	Weight%	Atomic%	Net int.	*K*-Ratio	*Z*	*A*	*F*
O K	38.14	64.86	60.77	0.0466	1.1443	0.1067	1.0000
Ti K	61.86	35.14	574.09	0.5626	0.9001	1.0079	1.0023

**Table 2:** eZAF SmartQuant results for MnO_2_@TiO_2_

Element	Weight%	Atomic%	Net int.	*K*-Ratio	*Z*	*A*	*F*
O K	27.09	53.65	53.32	0.0411	1.1817	0.1284	1.0000
Ti K	50.72	33.55	489.56	0.4825	0.9322	1.0008	1.0196
Mn K	22.19	12.80	124.51	0.1885	0.9050	0.9324	1.0064

According to the IUPAC guidelines, the N_2_ adsorption–desorption isotherm of the TiO_2_ NP definitively classifies it as a mesoporous material, as it exhibits a characteristic type IV profile with a well-defined H2 type hysteresis loop in the relative pressure range *P*/*P*_0_ = 0.4–0.8, as shown in Fig. 5(a). Interconnected mesopores with restricted desorption pathways specifically indicate the presence of the H2-type hysteresis loop. A dominant pore volume concentrated at a width of 2–8 nm is consistently shown by the BJH pore size distribution, with a sharp peak at approximately 3–5 nm, as revealed by the desorption curve.^33^ A mesoporous structure with a primary pore diameter of 3–5 nm is collectively and clearly established by these results to exist in the TiO_2_ NPs, as shown in Fig. 5(b) and (c).

Fig. 5(d) displays a type III N_2_ adsorption–desorption isotherm for MnO_2_@TiO_2_ NCs, which exhibits very low nitrogen uptake at low relative pressure, a sharp uptake near *P*/*P*_0_ = 1.0 and minimal hysteresis. Materials with weak adsorbate–adsorbent interactions and relatively low mesoporosity exhibit this isotherm type, which is consistent with the predominantly microporous or non-porous character of MnO_2_@TiO_2_ NCs, as shown in Fig. 5(d). We determined the BET surface area of MnO_2_@TiO_2_ NCs to be 102.4 m^2^ g^−1^ (adsorption) and 136.3 m^2^ g^−1^ (desorption).^34^ These results indicate a moderately high surface area despite the low mesoporous volume, which we attribute to surface roughness and small primary particle size rather than internal porosity. A dominant pore distribution at 2–4 nm is shown by the BJH desorption analysis of MnO_2_@TiO_2_ NCs (Fig. 5(e) and (f)),^35^ confirming that mesoporosity in this size range is retained by the composite. An enhanced accessible surface area is created by the combination of TiO_2_ mesoporosity and MnO_2_ surface area, which provides abundant active sites for dye molecule adsorption and subsequent photocatalytic degradation.^36^

### 3.4. Electrochemical and charge transfer characterization

**Fig. 6:**
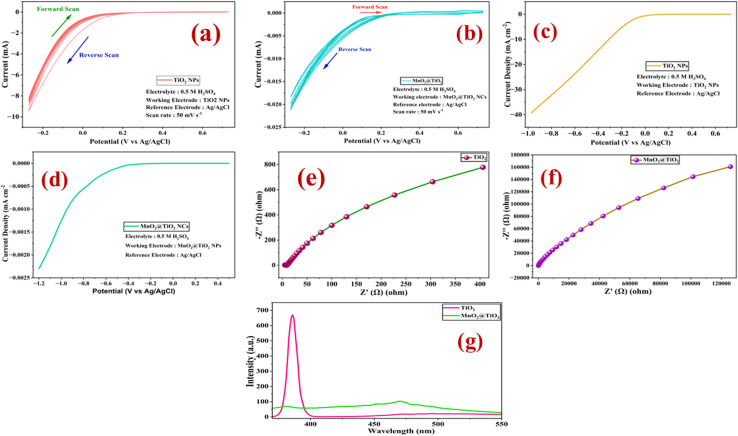
Electrochemical and optical characterization. CV profiles of the (a) TiO_2_ NPs and (b) MnO_2_@TiO_2_ NCs in 0.5 M H_2_SO_4_. LSV curves for the HER of the (c) TiO_2_ NPs and (d) MnO_2_@TiO_2_ NCs. Nyquist plots of the (e) TiO_2_ NPs and (f) MnO_2_@TiO_2_ NCs. (g) Photoluminescence spectra of the TiO_2_ NPs and MnO_2_@TiO_2_ NCs.

The cyclic voltammetry observations revealed that TiO_2_ in the H_2_SO_4_ electrolyte exhibit an unstable electrochemical response with a large inter-cycle variation, whereas the MnO_2_@TiO_2_ NCs show an improved cycle-to-cycle reproducibility and surface stability. This electrochemical stabilization indicated that the reactive surface of TiO_2_ is passivated by MnO_2_ deposition, which reduces unreactive surface reactions and creates a more chemically durable electrode–electrolyte interface. In the case of photocatalytic degradation, the stability of the surface directly connects to the sustained generation and utilization of reactive oxygen species (ROS) during the degradation of dyes and the stable surface ensures consistent photocatalytic activity over repeated reaction cycles, as shown in Fig. 6(a) and (b).

The modified surface chemistry of the composite was further confirmed by the LSV polarization for the HER. A large cathodic current density was exhibited by bare TiO_2_ NPs, reflecting high electrochemical reactivity and also indicating that non-selective electron consumption occurred at the electrode surface.^37^ In contrast, a substantially reduced HER current was shown by MnO_2_@TiO_2_ NCs, suggesting that photogenerated electrons are redirected by the MnO_2_ overlayer away from direct proton reduction and towards the other surface-mediated processes, which generates superoxide radicals (˙O_2_^−^) and other reactive species relevant to the photocatalysis oxidative degradation of organic dyes, as shown in Fig. 6(c) and (d).

A significantly higher charge transfer resistance (*R*_ct_) of MnO_2_@TiO_2_ NCs than that of bare TiO_2_ NPs was directly evidenced by the EIS Nyquist plots, and impedance values approximately two to three orders of magnitude greater are exhibited by the composite. While this may appear unexpected for a superior photocatalyst, it is important to identify that the materials' intrinsic electronic resistance is reflexed by the dark-state impedance in the absence of photoexcitation. Under visible light illumination, the effective interfacial resistance is substantially reduced by photogenerated carriers, which increase the surface carrier density. The insulating character of MnO_2_ itself therefore reflects the high dark state *R*_ct_ of MnO_2_@TiO_2_ NCs, and this character does not prevent and may actually support efficient photoinduced charge separation at the MnO_2_–TiO_2_ heterojunction under illumination, as shown in Fig. 6(e) and (f).

The most direct and conclusive evidence for the photocatalytic activity of MnO_2_@TiO_2_ NCs is provided by the PL spectroscopy results. This electron–hole recombination is drastically reduced by MnO_2_ integration, as demonstrated by the near-complete suppression of the TiO_2_ near-band edge emission peak in the composite, compared to the intense recombination fluorescence of bare TiO_2_ NPs. Photogenerated holes and electrons generate hydroxyl radicals (˙OH) and superoxide radicals (˙O_2_^−^), which then drive photocatalytic dye degradation.^38^ Higher steady-state concentrations of these reactive species and consequently greater dye degradation efficiency under visible light result directly from the prolonged carrier lifetime afforded by suppressed recombination in the MnO_2_@TiO_2_ NCs, as shown in Fig. 6(g).

Taken together, these findings establish that the interactive interplay between MnO_2_ and TiO_2_ at their heterojunction interface drives the enhanced photocatalytic performance of biologically synthesized MnO_2_@TiO_2_ NCs. Simultaneously acting as a surface stabilizer, a hole-trapping center, a visible-light sensitizer, and a recombination suppressor, MnO_2_ collectively enables more efficient utilization of incident photons for the photocatalytic degradation of organic dye pollutants. Using flower extracts in the green synthesis approach further ensures the formation of a well-dispersed, defect-rich MnO_2_@TiO_2_ interface that maximizes these synergistic effects, which underscores the dual advantages of the composite design and the sustainable synthesis strategy employed in the work.^39^

### 3.5. Antioxidant and antibacterial studies

The analysis for the antioxidant activity of TiO_2_ NPs and MnO_2_@TiO_2_ NCs was conducted at 5 mL concentration. At this concentration, TiO_2_ NPs exhibit 55.28% and MnO_2_@TiO_2_ NCs exhibit 75.92%, as shown in Fig. 6. The results show that the MnO_2_@TiO_2_ NCs show a good antioxidant activity in comparison to TiO_2_ NPs due to their enhanced molecular structure and chemical properties. This happens because MnO_2_@TiO_2_ NCs have more active sites for scavenging the free radicals than TiO_2_ NPs.

For determining the antibacterial activity of MnO_2_@TiO_2_ NCs and TiO_2_ NPs, a concentration of 5 µg mL^−1^ is taken, and it shows a zone of inhibition of 8 mm to 412 mm, which indicates that they have a good antibacterial activity, as given in Fig. 6.

**Fig. 7:**
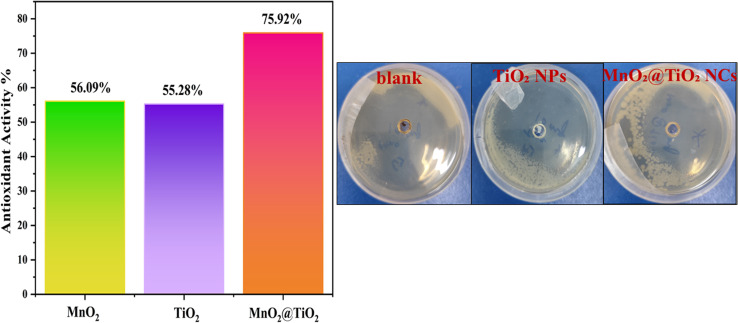
(Left) Antioxidant activity (%) of MnO_2_, TiO_2_ NPs, and MnO_2_@TiO_2_ NCs. (Right) Antibacterial activity showing the zones of inhibition for blank, TiO_2_ NPs, and MnO_2_@TiO_2_ NCs.

The greater the inhibition zone, the greater will be the availability of reactive surface sites. A good antibacterial activity shows that the process will increase ROS. These results suggest that the nanomaterials possess intrinsic antibacterial potential, which is amplified at increased concentrations, and these properties make them promising candidates for antimicrobial applications in the photocatalytic degradation of dyes (Fig. 7).

### 3.6. Photocatalytic degradation of MB and MO dyes

**Fig. 8:**
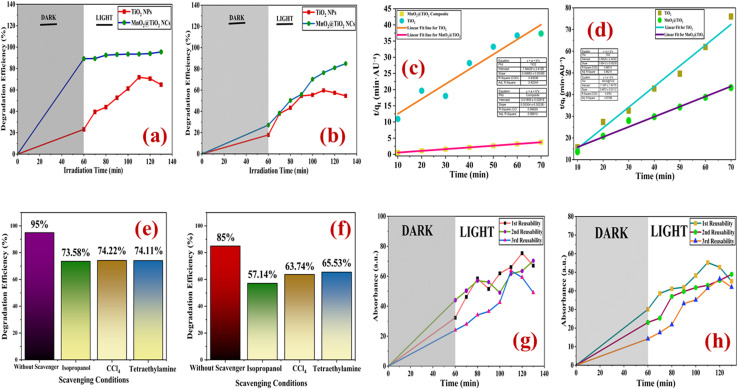
Photacatalytic performance. Degradation efficiency of (a) MB and (b) MO by the TiO_2_ NPs and MnO_2_@TiO_2_ NCs under dark and visible-light irradiation. Second-order kinetic plots for (c) MB and (d) MO degradation. Scavenger experiments for (e) MB and (f) MO. Reusability of the MnO_2_@TiO_2_ NCs over three cycles for (g) MB and (h) MO.

For MB degradation (Fig. 8), approximately 89% removal was achieved by MnO_2_@TiO_2_ NCs during the 60 min dark phase alone with only an additional improvement of ∼6% reached upon visible light irradiation for a total efficiency of 95%. We clearly state that this unusually high dark phase removal is due to the adsorption of the cationic MB dye onto the composite surface, rather than photocatalytic degradation. Hence, its photocatalytic activity is not considered.^40^ The high surface area, mesoporous structure of the MnO_2_@TiO_2_ NCs established by BET analysis and the experimental pH conditions drive this behaviour, which results from the strong electrostatic affinity between the positively charged MB molecule and the negatively charged metal oxide surface. Thus, the high adsorption capacity of MnO_2_@TiO_2_ NCs for MB is considered a surface property of the composite rather than a photocatalytic property, and the two contributions are clearly differentiated in this study, as shown in Fig. 8(a).

In contrast, MnO_2_@TiO_2_ NCs achieved a significantly lower dark phase removal of MO (Fig. 8(b)) at ∼26%. However, they drove the majority of degradation during the light irradiation phase, reaching ∼85%.^41^ Their contrasting molecular charges cause these differences between MB and MO behaviours, which are mechanistically significant. Strong electrostatic adsorption affinity for the negatively charged oxide surface is shown by the cationic dye MB. Conversely, electrostatic repulsion is experienced by the anionic dye MO from the same surface, resulting in lower adsorption and a greater proportion of true photocatalytic degradation. Therefore, the improvement for MO from ∼26% (dark) to ∼85% (light) indicates a genuine photocatalytic activity of ∼59%, which is directly caused by visible-light-driven reactive oxygen species generation. Complementary and self-consistent evidence for the photocatalytic mechanism is provided by this clear difference between adsorption-dominated MB removal and photocatalysis-dominated MO degradation, as shown in Fig. 8(b). Additionally, we compared the synthesised eco-friendly MnO_2_@TiO_2_ NCs with other previously reported catalysts for dye degradation. This shows a superior performance compared to the previously reported ones. Many catalysts rely on UV irradiation for their photocatalytic degradation but MnO_2_@TiO_2_ NCs works under visible light conditions, which provide sustainability along with solar applicability. The catalyst degraded methylene blue to approx. 95% within 70 min as compared to other catalysts that have achieved this degradation percentage in higher irradiation times, *i.e.* 150–300 min, as shown in Table 3.

**Table 3:** Comparison of the eco-friendly synthesized MnO_2_@TiO_2_ nanocomposites with previously reported photocatalysts for dye degradation

Nanocatalyst	Pollutant	Light source	Catalyst dose/mg	Dye conc./ppm	Irradiation time/min	Performance%	Ref.
Fe_3_O_4_/SiO_2_/TiO_2_	Methyl orange	UV-visible	20 mg	9.6 ppm	150	71.2	42
Fe_3_O_4_/SiO_2_/TiO_2_–Ag nanocomposite	Methylene blue	UV-visible	30 mg	50 ppm	180 min	80	43
Core–shell Fe_3_O_4_/SiO_2_/TiO_2_ nanospheres	Methyl orange	UV-visible	25 mg	30 ppm	5 h	90	43
TiO_2_/g-C_3_N_4_ nanocomposite	Methylene blue	Visible light	50 mg	10 ppm	150 min	92	44
g-C_3_N_4_/TiO_2_	Methylene blue	Visible light	2.3 g cm^−3^	10 ppm	240 min	97.6	45
CuO@SiO_2_	Methyl orange	Visible light	50 mg	20 ppm	40 min	79.79	46
CuO@SiO_2_	Crystal violet	Visible light	50 mg	20 ppm	40 min	94.27	46
SiO_2_@BiOBr	Methyl red	Visible light	25 mg	15 ppm	100 min	69	47
MnO_2_@TiO_2_ nanocomposite	Methylene blue	Visible light	50 mg	20 ppm	70 min	95.51	Present work
MnO_2_@TiO_2_ nanocomposite	Methyl orange	Visible light	50 mg	20 ppm	70 min	89.20	Present work

For MB degradation (Fig. 8(c)), an excellent fit for both materials is provided by the second-order model, with *R*^2^ = 0.99929 for MnO_2_@TiO_2_ NCs and *R*^2^ = 0.93536 for TiO_2_ NPs, which confirms the applicability of the second-order kinetic model to this system. The composite's substantially faster degradation kinetics is demonstrated by its second-order rate constant (slope = 0.053 min AU^−1^), which is roughly 8.7 times lower than that of TiO_2_ NPs (slope = 0.459 min AU^−1^), and this lower slope in the *t*/*q*_*t*_ plot indicates a higher degradation rate constant *k*_2_. For MO degradation (Fig. 8(d)), second-order kinetics were followed by both materials with high linearity, *R*^2^ = 0.9783 for MnO_2_@TiO_2_ and *R*^2^ = 0.9852 for TiO_2_ NPs. A lower slope (0.467) was displayed by MnO_2_@TiO_2_ NCs compared to TiO_2_ NPs (0.954), confirming approximately 2 times faster degradation kinetics for the composite toward MO. The photocatalytic enhancement achieved through the MnO_2_ surface modified is quantitatively confirmed for both dyes by the consistently superior kinetic performance of the MnO_2_@TiO_2_ NCs, as shown in Fig. 8(c) and (d).

For determining the identity and relative contribution of reactive oxygen species (ROS) responsible for photocatalytic dye degradation through scavenger experiments, specific chemical scavengers were introduced to selectively quench individual ROS. We used isopropanol (IPA) as a ˙OH scavenger, carbon tetrachloride (CCl_4_) as a ˙O_2_^−^ scavenger, and tetraethylamine as a photogenerated hole (h^+^) scavenger. The degradation efficiency of MnO_2_@TiO_2_ NCs under each scavenging condition is shown in Fig. 8(e) and (f).

For MB degradation (Fig. 8(e)), the addition of IPA reduced the efficiency from 95% to 73.58%, CCl_4_ reduced it to 74.22% and tetraethylamine reduced it to 74.11%. MB degradation is inhibited by approximately 21%, almost identically across the three scavengers. This indicates that ˙OH, ˙O_2_^−^, and h^+^ all contribute equally, with no single species being dominant. The type II heterojunction charge separation mechanism drives this near-equal contribution of all the three ROS, the holes accumulate on MnO_2_ to participate in direct oxidation and generate ˙OH, while the electrons on TiO_2_ produce ˙O_2_^−^.

For MO degradation (Fig. 8(f)), the addition of IPA reduced the efficiency from 85% to 57.14%, compared to CCl_4_ and tetraethylamine. OH is identified as the primary dominant reactive species for MO photodegradation by this greater suppression by IPA, with secondary roles played by O_2_^−^, and h^+^. The anionic character of MO causes the stronger ˙OH dependence of MO degradation than MB. The dominant reactive species and mechanistic pathways are clearly identified by these scavenger data, which quantitatively analyse both dyes.

The practical reusability of MnO_2_@TiO_2_ NCs was evaluated over three successive degradation cycles for both MB and MO. Significant degradation activity is maintained by the composite over three reuse cycles, although a gradual decrease in the maximum absorbance response is observed from the 1st to the 3rd reuse cycle, as shown in Fig. 8(g) and (h). This small decrease in catalytic activity over repeated use is assigned to partial surface site occupancy and incomplete dye desorption between cycles or less leaching of surface MnO_2_ species. Similarly, the catalyst retains considerable activity across three cycles for MO degradation, although it shows a slightly reduced response in the 3rd cycle compared to 1st. The MnO_2_@TiO_2_ NCs are recyclable photocatalysts with acceptable stability over at least three cycles, which is confirmed by these reusability results, supporting their practical applicability for water treatment. The structural and chemical stability of the composite under repeated operational conditions is independently confirmed by the CV data, which demonstrate a stable electrochemical response over 10 consecutive cycles in the same electrolyte.

**Fig. 9:**
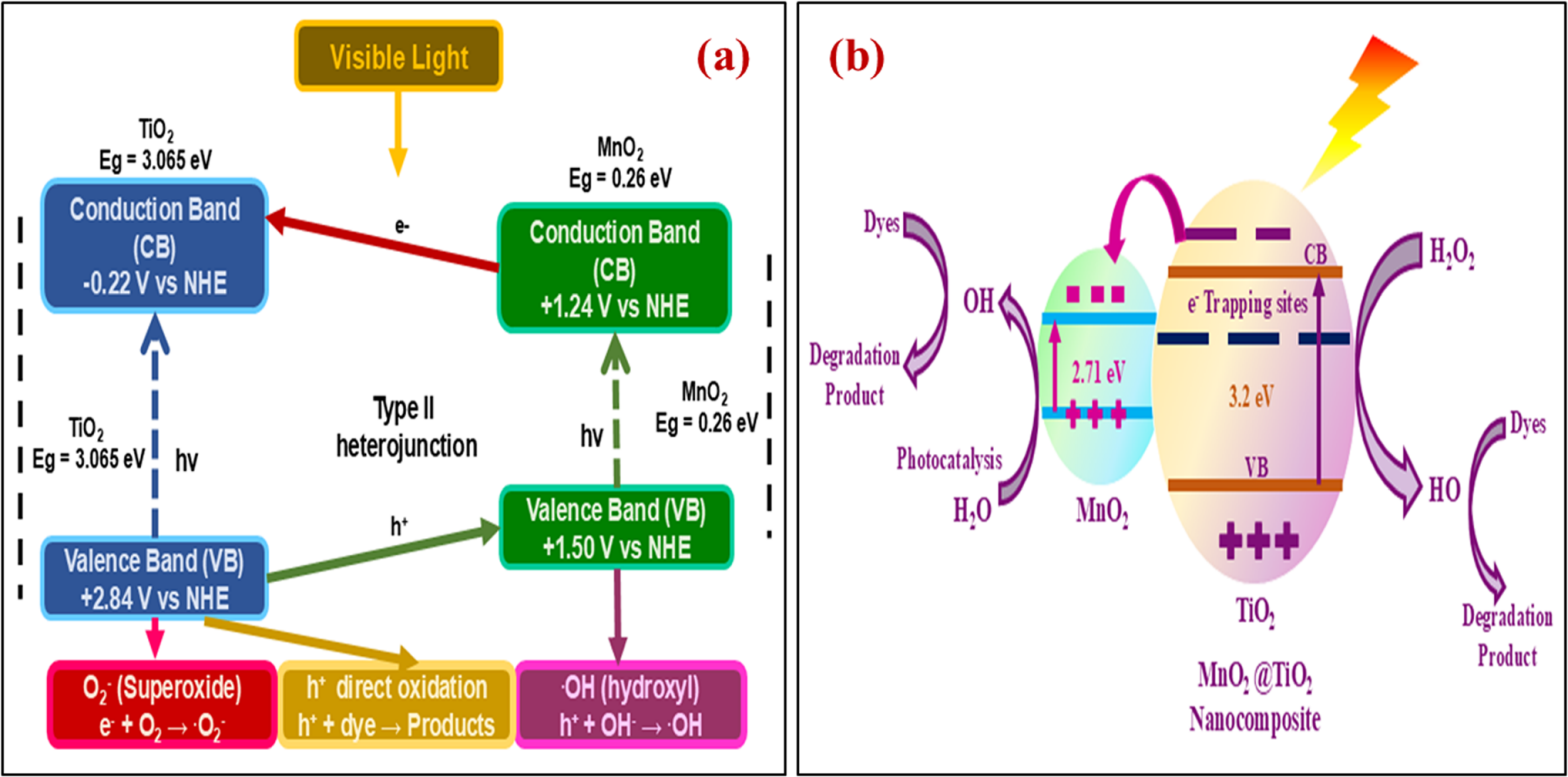
Proposed photocatalytic mechanism of the MnO_2_@TiO_2_ NCs under visible light irradiation. (a) Type II heterojunction band alignment showing the charge carrier separation and ROS generation pathways. (b) Schematic of the MnO_2_@TiO_2_ NC photocatalytic process.

Fig. 9 illustrates the photocatalytic mechanism of MnO_2_@TiO_2_ NCs under visible light irradiation. Using the Mulliken electronegativity approach, we estimated the CB and VB positions of TiO_2_ at −0.22 V and +2.48 V *vs.* NHE, while MnO_2_ exhibited CB and VB positions at +1.24 V and +1.50 V *vs.* NHE. A type II heterojunction is constituted by this shifted band alignment, thermodynamically driving the spatial separation of photogenerated charge carriers (with electrons moving to the TiO_2_ CB and holes to the MnO_2_ VB). This positional separation is directly confirmed by the 90% suppression of PL emission in MnO_2_@TiO_2_ NCs relative to pure TiO_2_ NPs.^48^

Under visible light irradiation (*E*_g_ = 2.95 eV, *λ* < 420 nm), dissolved O_2_ is reduced to superoxide radicals (e^−^ + O_2_ → ˙O_2_^−^) by separated electrons on the TiO_2_ CB, while surface hydroxyl groups are oxidized to hydroxyl radicals (h^+^ + OH^−^ → ˙OH) by holes on the MnO_2_ VB, which also simultaneously oxidize adsorbed dye molecules. All three species were experimentally confirmed by scavenger experiments.^49^

For MB (cationic), the equal contribution of all three ROS (˙OH = 21.4%, ˙O_2_^−^ = 20.8% and h^+^ = 20.9%) is facilitated by strong surface adsorption, which leads to the cleavage of the thiazine ring and its complete degradation into CO_2_, H_2_O, SO_4_^2−^, NO_3_^−^, and Cl^−^. For MO (anionic), electrostatic repulsion keeps it in solution, making ˙OH the dominant species (isopropanol = 27.86%), predominantly cleaving the azo bond (–N=N–) and producing CO_2_, H_2_O, SO_4_^2−^, and NO_3_ as final products.^50^ The overall reactions are summarized as follows:MnO_2_@TiO_2_ NCs + *hν* → e^−^_CB (TiO_2_ NPs) + h^+^_VB (MnO_2_),e^−^_CB + O_2_ → ˙O_2_^−^; h^+^_VB + OH^−^ → ˙OH,˙OH/O_2_^−^/h^+^ + MB/MO → CO_2_ + H_2_O + inorganic ions.

## 4. Conclusion

In this study, we successfully synthesised TiO_2_ NPs and MnO_2_@TiO_2_ NCs *via* a green sol–gel synthesis route using *Hibiscus rosa-sinensis* flower extract as a multifunctional reducing, capping and stabilizing agent. The phenolic contents of the extract simultaneously co-chelated the Ti^4+^ and Mn^2+^ precursors, promoting intimate nanoscale integration of both phases and producing verified Mn–O–Ti surface bonding (confirmed by FTIR spectroscopy), which is structurally essential for the observed photocatalytic enhancement. Phase-pure anatase TiO_2_ NPs (JCPDS 21-1272) with a crystallite size of 7.36 nm were prepared, as confirmed by PXRD analysis, which increased to 8.28 nm in the composite alongside β = MnO_2_ crystallites of 12.111 nm (JCPDS 24-0735). A systematic decrease in the (101) *d*-spacing of TiO_2_ NPs from 3.5200 Å provided direct crystallographic proof of MnO_2_–TiO_2_ surface lattice stress. SEM and TEM confirmed the surface modification, while BET established the mesoporous character of TiO_2_ NPs with a primary pore diameter of 3–5 nm. EDAX confirmed MnO_2_ surface modification rather than substitutional doping. A band gap narrowing from 3.065 eV to 2.95 eV was revealed by UV-DRS and Tauc plot analysis upon MnO_2_ incorporation, allowing visible light photoexcitation to be enabled. Approximately 90% suppression of electron–hole recombination was demonstrated by PL spectroscopy in MnO_2_@TiO_2_ NCs, confirming efficient charge separation at the type II heterojunction. The modified interfacial charge transfer dynamics and superior electrochemical stability of the composite were further confirmed by EIS and CV. Under visible light irradiation, MnO_2_@TiO_2_ NCs achieved degradation efficiencies of 95% for methylene blue and 85% for methyl orange, significantly outperforming pure TiO_2_ NPs, which only reached 65% and 55%, respectively. The MB degradation kinetics was confirmed by second-order kinetic modelling to be approximately 8.7 times faster. Scavenger experiments identified ˙OH, ˙O_2_^−^, and h^+^ as the active-reactive oxygen species, and they identified ˙OH as the dominant species for MO degradation. The practical stability of the composite was confirmed by catalyst reusability over three successive cycles. Overall, MnO_2_@TiO_2_ NCs are established as highly effective, recyclable, and sustainably synthesized visible-light photocatalysts with promising applicability in water purification technologies by the synergistic interplay of band gap narrowing, suppressed charge recombination, mesoporous surface modification and MnO_2_-mediated hole trapping. An environment-friendly, cost-effective and sustainable approach is provided by the green synthesis method as well as scalable substitute to conventional methods; while relaying antioxidant as well as antibacterial properties obtained from the hibiscus extract. Collectively, these results confirm that the phyto-fabricated MnO_2_@TiO_2_ NCs are multifunctional materials with great promise for sustainable wastewater treatment and potential applications in antibacterial coatings, wound care, and other environmental remediation techniques.

## Author's contributions

Yashneeti Mehta conceptualized the study, designed and conducted all the experiments, and optimized the experimental parameters. Sonal Chauhan performed the bacteriological activity analysis. Dinesh Kumar Arya carried out the material characterization. Nitesh Kumar contributed to the characterization work and revised the manuscript for technical accuracy. Sheenam Thatai and Parul Khurrana reviewed and revised the draft and contributed to the finalization of the manuscript. All authors have read and approved the final version of the manuscript for submission.

## Conflicts of interest

The authors declare that they have no competing interests.

## Abbreviations

MnO_2_: Manganese dioxide
TiO_2_: Titanium dioxide
MnO_2_@TiO_2_: Manganese doped titania
NPs: Nanoparticles
NCs: Nanocomposites
MnSO_4_·H_2_O: Manganese sulfate monohydrate
TEOT: Tetraethyl orthotitanate
UV-vis: Ultraviolet-visible spectroscopy
DLS: Dynamic light scattering
FTIR: Fourier transform infrared spectroscopy
XRD: X-ray diffraction
SEM: Scanning electron microscopy
TEM: Transmission electron microscopy
BET: Brunauer–Emmett–Teller
BJH: Barrett–Joyner–Halenda
MO: Methyl orange
MB: Methylene blue
PL: Photoluminescence spectroscopy
CV: Cyclic voltammetry
LSV: Linear sweep voltammetry
NHE: Normal hydrogen electrode
CB: Conduction band
VB: Valence band

## Data Availability

The data supporting this article, including the UV-vis and FTIR spectra, XRD patterns, SEM/TEM images, BET analysis, and photocatalytic degradation data, have been included within the article.
